# Gendered reacculturation and marriage negotiation among single Chinese female returnees

**DOI:** 10.1186/s12889-026-28227-x

**Published:** 2026-06-23

**Authors:** Ruining Jin, Boya Wu, Jie Wei, Xiao Wang

**Affiliations:** 1 Institute of Higher Education, Beijing University of Technology, Beijing, 100124 China; 2 Civil, Commercial and Economic Law School, China University of Political Science and Law, Beijing, 100088 China; 3 Department of Psychology, University of Macau, Taipa, Macau 519000 China

**Keywords:** Reacculturation, Single women, Gender studies, Return migration

## Abstract

**Background:**

Returning home after international study can involve complex readjustment, especially when personal life plans, family expectations, workplace experiences, and broader social norms do not fully align. In contemporary China, never-married female returnees may encounter distinctive expectations concerning marriage timing, family responsibility, career development, and future life planning.

**Method:**

Drawing on Bronfenbrenner’s Ecological Systems Theory and Patriarchal Bargaining, this study adopts a qualitative design based on in-depth semi-structured interviews with 22 never-married Chinese female returnees who had studied in Western countries and returned to China.

**Findings:**

The findings suggest that participants’ post-return experiences were shaped by multiple, interconnected layers of social life. At the family level, they encountered expectations related to marriage timing, filial responsibility, and socially approved life-course choices. At the workplace level, they described gendered assumptions, unequal professional opportunities, and concerns about career mobility. At the broader societal level, participants perceived public discussion around unmarried women and women with overseas experience as an additional source of pressure. These experiences shaped how participants evaluated marriage, with many approaching it cautiously while weighing family expectations, institutional conditions, reproductive concerns, and future quality of life.

**Implications:**

The study suggests that continued singlehood among female returnees should not be understood simply as individual preference or delayed transition. Rather, it can reflect an ongoing negotiation of gendered expectations during reacculturation. The study contributes to scholarship on return migration, singlehood, and gender by showing how family, workplace, and wider sociocultural contexts intersect in shaping women’s post-return lives in contemporary China.

**Supplementary Information:**

The online version contains supplementary material available at https://doi.org/10.1186/s12889-026-28227-x.

## Introduction

In an era of intensified global mobility, the return of internationally educated migrants has become an increasingly important concern in migration and intercultural studies [1–5]. Yet returning “home” is rarely a simple reversal of outward mobility. After extended exposure to different cultural norms, institutional environments, and social expectations abroad, returnees often undergo a process of reacculturation in which they must renegotiate identity, relationships, and everyday practices within their home society [6, 7]. Existing studies have shown that this process may involve sociocultural dissonance, identity conflict, and psychological strain, especially when returnees perceive a mismatch between the values and life orientations developed overseas and the expectations they encounter upon return [8–10]. In post-COVID-19 China, these challenges may be further intensified by broader economic uncertainty, labor-market pressure, and renewed public anxiety surrounding marriage, fertility, family formation, escalating Sino-West tension, and the global rise of right-wing conservative movements [11–17].

More importantly, reacculturation is not gender-neutral, as women may experience reintegration through a distinctly gendered set of expectations shaped by family roles, workplace hierarchies, and broader cultural understandings of femininity [18–20]. In this study, femininity is understood as a set of expectations concerning how women are supposed to behave as daughters, workers, intimate partners, and potential wives or mothers [21–23]. In the Chinese context examined here, these expectations include timely marriage, sexual respectability, filial responsiveness, and other life-course choices that are seen as socially respectable [24–29]. Earlier research has suggested that factors such as marriage, caregiving, and family obligation, and gendered expectations surrounding women’s conduct may intensify cross-cultural strain, making return and reintegration especially complex for women in some contexts [4, 18, 19, 30–32]. This gendered dimension is particularly salient in the Chinese context, where single professional women have already been the subject of substantial feminist scholarship. Studies on *sheng nü* (“leftover women”) have shown that highly educated unmarried women in urban China are often positioned at the intersection of contradictory expectations: they are encouraged to be modern, accomplished, and competitive, yet are also expected to marry within a socially appropriate timetable and uphold conventional gender norms [24, 26]. Delayed marriage is therefore not treated as a neutral demographic condition, but as a socially and morally charged status associated with parental pressure, partner-market constraints, and stigmatizing public discourse [25, 27].

However, compared with domestic unmarried professional women, single female returnees may face an additional layer of suspicion because their unmarried status could be interpreted not only through patriarchal norms, but also through anxieties about Westernization, foreign ties, and national-cultural loyalty. Because of their overseas experience, female returnees possess a transnational identity [33]. Although this identity may afford bilingual competence, wider social networks, and cross-border mobility, it may also expose them to added psychological strain and suspicion in post-COVID-19 China [4, 34], especially in a context in which nationalist sentiment has remained highly salient and has been reshaped through crisis politics, online discourse, and intensified concern with cultural boundaries [35, 36]. This matters for female returnees because nationalism is not gender-neutral. Research on China’s nationalist discourse points to its enduring gender undertone, in which women are often symbolically aligned with cultural authenticity, moral order, and collective loyalty rather than treated simply as autonomous individuals [37–39]. Under this form of gendered nationalism, women’s bodies, sexuality, and intimate choices may be treated as matters of national dignity and cultural belonging. One recent study in 2025 specifically suggests that Chinese women are often stigmatized, facing societal judgments when dating people from other racial groups in China [28].

Recent public controversies also illustrate this additional layer of pressure faced by female returnees. In 2025, it was reported that a Chinese university expelled a female student for allegedly “damaging national dignity” through “improper interactions” with a foreign man [40]. Also in 2025, a female Chinese international student in the United States was murdered during her overseas study [41, 42]. However, Chinese diaspora media, domestic media, and social media focused mainly on the sexual intimacy and the leaked footage between the deceased student and her white boyfriend [43, 44], rather than the loss of a young life. More recently, Chinese influencer *Lao A*, who drew attention for popularizing the phrase *“kill line”* in commentary on social decline of American Middle Class in the United States [45, 46], later generated controversy over misogynistic remarks about Chinese women living or studying abroad, including sexually derogatory insinuations about their morality and their relationships with Western men [47]. Taken together, these incidents reinforce the present study’s argument that female returnees face an additional layer of pressure during reacculturation, as their transnational identities and intimate lives may be judged through both gendered expectations and concerns about cultural loyalty beyond the marriage-related stigma already documented among domestic single women.

### Theoretical framework

#### Ecological systems theory

Bronfenbrenner’s Ecological Systems Theory (EST) provides a useful framework for understanding reacculturation as a process shaped by multiple, interrelated layers of social life rather than by individual adjustment alone [48]. This perspective is especially appropriate for the present study because the experiences of single female returnees are not confined to one domain. Instead, their difficulties emerge across interconnected settings, including family relationships, institutional environments, and broader sociocultural discourse. EST therefore allows the study to move beyond intrapersonal stress and examine how reacculturative pressures are structured across layered contexts.

#### Patriarchal bargaining

While EST helps explain where pressures originate, it does not by itself explain how women interpret marriage and singlehood within this gendered environment. To address this dimension, the present study draws on the concept of Patriarchal Bargaining (PB) [49]. PB begins from the premise that women do not make life decisions within a socially neutral or fully equal landscape. Rather, they navigate gender orders in which risks, obligations, and rewards are distributed unequally, and they may comply with, negotiate, delay, selectively resist, or refuse particular expectations in order to secure the most livable position available to them.

This framework is especially useful for the present study because it conceptualizes marriage not as a purely private or individual matter, but as a social institution embedded in broader structures of gender hierarchy. In this sense, women’s orientations toward marriage and singlehood are shaped not only by personal preference, but also by culturally specific expectations concerning femininity, family obligation, reproductive roles, and the distribution of authority and responsibility within intimate life. Patriarchal Bargaining therefore provides a suitable lens for examining how women position themselves in relation to marriage under conditions where the terms of participation may be perceived as unequal, restrictive, or insufficiently rewarding.

### Current study

Although prior scholarship has examined Chinese returnees’ reacculturation difficulties and the stigmatization of unmarried professional women in China, limited attention has been paid to the intersection of these two trajectories. Existing domestic feminist research has shown that highly educated unmarried women in urban China often negotiate intense pressure surrounding marriage timing, filial obligation, femininity, and social respectability [24–27]. Separately, returnee studies have demonstrated that post-return adaptation may involve sociocultural dissonance, identity conflict, perceived discrimination, and psychological strain, especially when overseas-acquired values come into tension with local norms and institutional realities [4, 5, 8, 9]. Yet less is known about how these processes converge for women who are both internationally educated returnees and unmarried in a social context where marriage remains an important sign that a woman is seen as following the expected life path.

The present study addresses this gap by examining the experiences of single Chinese female returnees in post-COVID-19 China. In this study, “single” refers to never-married overseas-educated returnee women who were unmarried at the time of interview. Rather than treating reacculturation stress and singlehood as separate themes, the study approaches them as analytically intertwined. For this population, reintegration does not occur in a social vacuum. Rather, it unfolds within a gendered environment in which family expectations, workplace hierarchies, and broader public discourses about women’s roles, marriage, and perceived national or cultural loyalty are simultaneously salient. As a result, the challenges these women face cannot be reduced either to generic returnee adjustment or to the domestic stigma already documented among unmarried professional women in China. Instead, they are better understood as part of a broader process of gendered reacculturation. Guided by this perspective, the present study asks two questions.

#### RQ1

What multi-layered pressures, challenges, and forms of psychological distress do single Chinese female returnees experience during reacculturation in post-COVID-19 China?

#### RQ2

How do these pressures shape the ways single Chinese female returnees interpret, negotiate, and respond to marriage-related expectations?

By focusing on the intersection of return migration, gendered reintegration, and singlehood, this study seeks to offer a more integrated account of how internationally educated women navigate post-return readjustment in contemporary China.

## Methodology

### Participants and data collection

This study adopted a phenomenologically informed qualitative design to examine how single Chinese female returnees understood and negotiated gendered reacculturation and marriage-related expectations after returning to China. A qualitative approach was appropriate because the study aimed to explore participants’ lived experiences, subjective meanings, and post-return interpretations across family, workplace, and broader sociocultural contexts.

The study focused on single Chinese female returnees who had studied in Western countries and were residing and working in China at the time of interview. In this study, “single” was defined by legal and marital status rather than by the complete absence of romantic relationships. Participants were eligible if they had never been legally married at the time of interview; being in a dating relationship did not automatically exclude participation. A purposive sampling strategy was used to recruit participants who met the following criteria: (1) they were born and raised in China; (2) they were aged 28 or older; (3) they had studied abroad for at least two years in North America, Europe, or Oceania; (4) they had returned to China and had lived in China for more than one year; and (5) they were never married at the time of interview. A total of 22 participants were recruited through the researchers’ personal networks and several WeChat returnee groups. Because access to this population often depends on trust, network entry points, and willingness to discuss sensitive topics, purposive recruitment through personal contacts and returnee groups was considered appropriate. At the same time, this means that the sample should be understood as network-accessed rather than statistically representative.

Participants ranged in age from 28 to 38 years and had studied in North America, Europe, or Oceania. They held bachelor’s, master’s, or doctoral degrees and worked across a range of occupations, including teaching, research, marketing, public relations, accounting, design, and public-sector or state-affiliated positions. These demographic characteristics are presented in (Table 1).

**Table 1:** List of participants

Name	Region of overseas study	Age	Degree	Years Abroad	Years Returned	Occupation
P1	Europe	31	Master	6	2	Teacher
P2	Oceania	28	Bachelor	4	6	Teacher
P3	Europe	29	Master	2	5	Teacher
P4	North America	33	Master	6	3	Overseas Marketing Director
P5	North America	35	Master	2	11	Account Manager
P6	North America	33	Doctor	10	3	Researcher
P7	North America	32	Doctor	9	4	Marketing Manager
P8	North America	28	Master	2	7	Teacher
P9	North America	29	Master	2	5	E-Gaming Public Relations
P10	Europe	30	Master	2	6	Social Media Operation Specialist
P11	Europe	32	Doctor	9	4	Social Media Operation Specialist
P12	North America	33	Master	2	4	Technician in a Public Institution
P13	North America	28	Master	2	5	Streamer
P14	North America	29	Bachelor	4	4	Public Relations Manager
P15	North America	38	Doctor	10	3	Fashion Designer
P16	North America	37	Master	2	4	Teacher
P17	Europe	31	Master	5	3	Human Resource Manager in a State-run Company
P18	North America	28	Master	3	1	Accountant in a State-run Company
P19	Oceania	32	Master	3	5	Teacher
P20	North America	33	Master	6	4	Marketing Specialist
P21	Europe	34	Doctor	8	5	Researcher
P22	North America	37	Doctor	10	4	Researcher

The research team consisted of four Chinese researchers, including three women and one man. Because the topic involved sensitive issues such as harassment, intimate relationships, family pressure, and marriage, all interviews were conducted only by the three female researchers. The male researcher did not participate in interviewing, but contributed to later coding, analytic discussion, and interpretation. The lead researcher held a doctorate and was employed full-time at a higher education institution during the study period. The remaining team members had also received doctoral-level research training. Several members of the team had themselves experienced transnational mobility, and some had encountered gendered reacculturation pressures in their own lives. These backgrounds helped facilitate rapport and contextual understanding during the interviews, while also requiring ongoing reflexive attention during analysis.

A limited prior relationship existed with some participants who were recruited through the researchers’ personal or professional networks, whereas no prior personal relationship had been established with participants approached through WeChat returnee groups. When contacting potential participants, the study was described broadly as research on the post-return experiences of single female returnees in China, including work adjustment, family relationships, everyday social life, and future planning. The recruitment message did not foreground harassment, anti-marriage attitudes, or highly negative experiences, so as to reduce the likelihood of recruiting only participants with particularly adverse accounts.

The main interview wave was conducted between September 15 and October 15, 2024. All interviews were carried out remotely through one-to-one WeChat voice calls, which allowed participation across different cities and provided a familiar and accessible setting for discussing sensitive experiences. No non-participants were reported to be present during the interviews. Each initial interview lasted approximately 30 to 60 min. All interviews were conducted in Mandarin Chinese to minimize linguistic constraint and to allow participants to express personal and culturally sensitive experiences with greater precision. A semi-structured interview guide, developed by the authors and refined through team discussion prior to formal data collection, was used to explore participants’ post-return adjustment, family expectations, workplace experiences, perceptions of broader public discourse, and reflections on marriage, fertility, and future life planning. The guide was not formally pilot-tested and is provided as supplementary material.

To capture newly emerging developments affecting female returnees in China, a second round of interviews was conducted between March 23 and March 30, 2026. This follow-up wave included 20 of the original 22 participants; two participants did not take part due to loss of contact or non-response. Each follow-up interview lasted approximately 30 to 45 min and was conducted remotely through one-to-one WeChat voice calls. The compressed timeline was feasible because the interviews were shorter than the first-wave interviews, distributed among the three female interviewers, and facilitated by rapport already established during the first wave. Recent public controversies concerning female returnees also made the topic timely and personally relevant, which increased participants’ willingness to re-engage with the study. The same interview guide was used in both waves, allowing the researchers to revisit earlier themes while also examining how gendered public controversies, online hostility, and intensified stigmatization of women with overseas experience shaped participants’ subsequent interpretations. Rather than constituting a separate dataset, the follow-up interviews were incorporated into the same analytic process as the first-wave interviews in order to refine, update, and extend the interpretation of themes already identified.

All interviews were audio-recorded with participants’ consent and transcribed in Mandarin Chinese. Brief field notes were made during and/or immediately after interviews to document contextual observations, emerging analytic reflections, and issues to revisit in later team discussions. Written informed consent was obtained via WeChat prior to the initial interview, and the original consent covered possible continued participation in the study, making renewed formal consent unnecessary at the follow-up stage. Participants’ confidentiality was protected through pseudonyms or participant codes. As the study involved human subjects, it underwent ethical review and received approval from the Institutional Review Board of China University of Political Science and Law.

### Data analysis

The interview transcripts were imported into MAXQDA for analysis using an abductive thematic approach, following established procedures for qualitative thematic analysis [47, 48]. This strategy was adopted because the study sought both to identify recurring patterns in participants’ accounts and to interpret those patterns in relation to broader theoretical concerns about gendered reacculturation and marriage negotiation. The analysis was also informed by a phenomenological interest in how participants themselves made sense of their lived post-return experiences.

All four researchers participated in the coding and analytic process. The analysis unfolded in several stages. First, the research team engaged in repeated close reading of the transcripts to become thoroughly familiar with the data and to identify recurring patterns in participants’ accounts of return, family relations, workplace experiences, public discourse, and marriage-related expectations. Second, an initial round of open inductive coding was conducted, allowing codes to emerge from participants’ narratives rather than being rigidly imposed in advance. Third, conceptually related codes were grouped into subthemes and broader themes through iterative comparison and team discussion. A provisional coding tree was developed to organize themes across major domains relevant to the research questions, including family pressure and kin obligations, workplace sexism and harassment, public discourse and gendered stigma, orientations toward marriage, reproductive concerns, and trust in men and intimate partnership. Fourth, these themes were interpreted in light of the study’s guiding frameworks, namely Ecological Systems Theory and Patriarchal Bargaining. In this sense, the research questions and theoretical frameworks functioned as sensitizing lenses, but the substantive themes themselves were derived from the data and refined through iterative interpretation.

The follow-up interviews were incorporated into the same coding and analytic process rather than treated as a separate dataset. Because the second wave revisited the same themes with 20 of the original participants, it enabled the research team to refine and update the analysis in response to newly emerging public and social dynamics, especially those concerning online misogyny, gendered public hostility, and female returnees’ trust in men and marriage. Thus, the follow-up interviews deepened the temporal and contextual sensitivity of the findings rather than generating an entirely new thematic structure.

Data collection and analysis proceeded iteratively. Recruitment and interviewing were brought to a close when the research team judged that the data provided sufficient depth to address the research questions and that no substantive new themes were emerging across the core domains under investigation. All interviews were transcribed in Mandarin Chinese. Quotations selected for presentation were first translated into English by a professional translator and then checked against the original Mandarin transcripts by a bilingual researcher. Where wording, tone, or culturally specific meanings were difficult to render directly, the team discussed the excerpt and revised the translation until agreement was reached. The goal was to preserve participants’ intended meaning, emotional tone, and culturally embedded expressions rather than to produce strictly literal translations. No AI-assisted translation or machine-translation tools were used.

### Trustworthiness and reflexivity

To enhance the credibility and analytical rigor of the study, several trustworthiness strategies were employed, including iterative coding, ongoing team debriefing, field-note writing, explicit attention to researcher positionality, and member-checking of selected quotations and emerging interpretations with participants. These procedures were intended not to claim positivist objectivity, but to strengthen transparency, coherence, and interpretive accountability throughout the research process.

The researchers recognized that their own biographies shaped both data collection and interpretation. As Chinese researchers with doctoral-level training, and with several members having personal experiences of transnational mobility, the team was well positioned to understand participants’ linguistic expressions, cultural references, and return-related dilemmas. In particular, the three female interviewers were able to build rapport when discussing sensitive issues such as harassment, stigma, bodily risk, marriage pressure, and women’s post-return vulnerability. At the same time, the team remained aware that experiential proximity can also create risks of over-identification, assumption, or selective attention. Reflexivity was therefore treated as an ongoing collective practice rather than a one-time declaration: the team held regular discussions to examine assumptions, compare alternative readings of the data, and revisit interpretations when necessary. The male researcher, although not involved in conducting interviews, contributed to coding and interpretive discussion, which also helped broaden analytic scrutiny and reduce the risk that shared assumptions among the female interviewers would go unquestioned.

Given the sensitivity of the topic, participants were reminded at the outset that they could decline to answer any question, pause the interview, or withdraw from the study at any time without negative consequences. Full transcripts were not returned to participants for line-by-line correction; however, selected quotations and developing interpretations were checked with participants during the analytic process, and participant feedback was incorporated where relevant to improve clarity and interpretive accuracy.

## Findings

### Layered pressures in gendered reacculturation

Participants described reacculturation as a multi-layered process of distress that unfolded across family relationships, workplace settings, and the broader public sphere. Their difficulties were not confined to internal readjustment alone. Instead, they emerged through repeated encounters with gendered expectations concerning marriage, kin obligation, professional conduct, and women’s social value.

#### Family pressure, boundary violations, and life-course expectations

At the mesosystem level, participants described family as one of the most immediate sites of reacculturative strain. Much of this pressure centered on marriage timing, women’s proper roles, and the expectation that daughters should align their life choices with family priorities rather than personal aspirations.

*“Every Lunar New Year*,* I dread going back to my hometown. It feels like a trial: my relatives gather like juries*,* my parents become judges*,* and my ‘crime’ is being a single woman living alone in another city.” (P22)*.

This familial pressure extended beyond marriage itself. Participants also described conflict over personal boundaries and family obligation, especially when they felt their own needs were subordinated to kinship expectations.

*“My younger cousin keeps living in my parents’ apartment and asking my father to use his connections to help him switch jobs. He is an adult and should handle his own life*,* but my parents say my way of thinking is too selfish.” (P7)*.

Related tensions also emerged around career choice and future planning. For some participants, return intensified conflict between their own aspirations and their parents’ preference for security, stability, and conventionally respectable occupations.

*“I love animation and want to devote my talent and passion to this industry*,* but my parents think it is unstable and not financially rewarding. They would rather I teach English or become a civil servant for the sake of stability.” (P14)*.

Taken together, these accounts show that family was not merely a source of support during reacculturation. It was also a central site where participants encountered pressure to conform to gendered expectations concerning marriage, duty, and respectable life-course choices.

#### Workplace sexualization, harassment, and restricted career mobility

At the exosystem level, participants described the workplace as another major source of distress. Return to China meant not only re-entering the labor market, but also re-entering professional environments in which women’s bodies, conduct, and advancement were unequally regulated.

Some participants described sexual harassment as pervasive in work settings marked by unequal power relations, particularly between supervisors and subordinates or between male clients and female employees.

*“A lot of the sexual harassment I experienced*,* or heard about from friends*,* happened in unequal relationships. It was usually between male supervisors and female employees*,* or between male clients and female agents.” (P10)*.

Others suggested that their overseas background could itself become sexualized in workplace interactions. In these cases, female returnees were not simply treated as employees, but as women presumed to be more sexually open because of their foreign experience.

*“At a business dinner*,* one man tried to hug me and kiss my face because he assumed that all female returnees from the U.S. were sexually open and used to that kind of greeting. When I asked where he got that idea*,* he said*,* ‘American Pie.’” (P6)*.

A similar dynamic appeared in less overtly physical but still clearly sexualized forms of workplace interaction. As P2 explained, her supervisor often made “dirty” or pornographic jokes in front of her and seemed to assume that, because she had overseas experience, she would be sexually open enough to accept such remarks and laugh along with them. Here, overseas education was not treated as a marker of expertise, but as a cue for sexualized assumptions about women’s boundaries.

Participants also emphasized that workplace inequality extended beyond harassment to professional advancement. For some, promotion was shadowed by sexual rumor and informal patronage, while senior positions remained structurally male-dominated.

*“The unspoken rule is that if a woman gets promoted*,* people assume it must be because someone powerful slept with her—or with her mother. That captures how parental connections*,* sexual harassment*,* and career ambition get entangled.” (P2)*.

*“In the companies where I worked*,* positions involving policymaking*,* administration*,* and major financial decisions were usually reserved for men. Women could only compete for a small number of senior roles seen as less important and paid less*,* such as HR or public relations.” (P5)*.

Overall, these accounts show that workplace reintegration was not experienced simply as occupational readjustment. It was shaped by recurrent exposure to harassment, sexualized stereotyping, and restricted mobility within gendered professional hierarchies.

#### Public hostility, moral judgment, and devaluation in the wider social sphere

At the macrosystem level, participants described distress not only as a matter of direct interpersonal experience, but also as something produced through wider public discourse. They perceived the broader social environment as one in which women were repeatedly judged, belittled, and morally scrutinized in media and everyday public culture.

Some participants pointed to routine media and social-media representations that treated women as inherently less competent or more blameworthy than men.

*“I’m tired of how society and the media stigmatize women. On social media*,* if there’s a traffic accident and the driver is a woman*,* the headline always points that out*,* as if it explains the accident. But when men make the same mistake*,* their gender is never mentioned.” (P1)*.

Public hostility intensified markedly when Chinese women’s transnational mobility became entangled with intimate relationships involving foreign men, revealing deeper currents of nationalist resentment and gendered stigma in domestic online discourse. P17 recalled in relation to the murder of a Chinese female student at an American university:

*“Much of the domestic online discussion I encountered focused less on the violence itself than on leaked images of the victim’s intimate life with her American boyfriend. I was particularly disturbed by comments suggesting that such cases explained why returnee or internationally mobile women would not marry Chinese men*,* with some users framing these women’s sexual preferences as deviant or ‘perverted.’”(P17)*.

For P17, this episode exemplified how female returnees and students abroad were recast, not as victims or autonomous individuals, but as objects of sexualized stigma and nationalist resentment.

Participants also described more direct forms of gendered devaluation in professional and public settings, where women’s appearance or femininity was used to undermine their credibility.

*“During a post-doc interview*,* the interviewer said*,* ‘You’re pretty—you could have chosen an easier career path. So why are you here?’ I found that unbearable and decided to apply somewhere else.” (P2)*.

In short, participants portrayed the broader social environment as one in which women were not only expected to marry, but also subjected to repeated public belittlement, moral judgment, and, in some cases, stigma focused on women’s sexuality. These macrosystemic pressures intensified reacculturation by making return feel like re-entry into a social world where women’s value remained highly conditional.

### Marriage as an unequal and risky arrangement

Building on the layered pressures identified in RQ1, participants’ narratives suggest that marriage was increasingly evaluated as an unequal bargain rather than a natural or desirable life stage. Their accounts did not indicate a simple rejection of intimacy or partnership. Instead, they revealed growing reluctance to enter a social arrangement perceived as offering limited protection, asymmetric obligations, and uncertain returns under current family, institutional, and public conditions.

#### Family pressure made marriage socially useful, but not worth unequal entry

At the family level, some participants recognized that marriage could reduce parental scrutiny and ease kin pressure. In some cases, marriage was also seen as potentially useful because it could connect women to additional family or kin-based resources. Yet these possible benefits were not treated as sufficient reasons to enter an unequal or low-quality relationship.

*“In China today*,* your personal ability always comes after your parental networks. So for me definitely I can go further in my workplace if there is additional parental support. Also*,* when I get married*,* I don’t need to be the defendant in family court facing those judges and juries*,* I guess. But this does not mean I have to endure a poor marriage because of their judgment. Honestly*,* I care more about how I feel rather than how they feel.” (P22)*.

Similar reflections appeared in other participants’ accounts: marriage was sometimes described as socially useful, but only conditionally acceptable if it did not require sacrificing autonomy, dignity, or emotional well-being.

*“I have a close friend who recently got married. With help from her parents-in-law*,* she got hired in a public institution. I’m kind of jealous of that part. But she also complained a lot about how exhausting a poor mother-in-law–daughter-in-law relationship could be. Because of that*,* I also feel quite fortunate.” (P2)*.

These accounts suggest that participants recognized that marriage could reduce family pressure and, in some cases, provide access to kin-based resources. However, they evaluated these benefits against the possible costs of unequal marital relations, emotional burden, and intensified family obligations.

#### Legal/sociocultural exit constraints made marriage riskier to enter

Some participants also interpreted marriage through the legal and sociocultural conditions surrounding marital exit. Their concerns were not limited to whether a partner might be suitable; they also focused on whether marriage, once entered, could be exited without excessive procedural difficulty or vulnerability. P20 offered her account of how local legal interpretations on marriage cool-off period made divorce costly.

*“In my city*,* I heard that when you want to file your divorce application*,* there is a 30-day cool-off period. During this stage*,* if you want to divorce but your husband doesn’t want to*,* the court won’t support your claim. Even if both of you agree*,* you need to make an appointment in advance*,* and they set very limited appointments available for divorce but unlimited for marriage. This is RIDICULOUS!” (P20)*.

Similarly, P15 emphasized the sociocultural costs women may face after leaving a marriage, especially when divorce is tied to motherhood and childrearing responsibility.

*“In China’s dating market*,* a woman’s value is often judged by whether she has married*,* divorced*,* or had children. Once a woman enters marriage*,* she faces enormous pressure to have a baby. But if she has a child and later divorces*,* especially with a son*,* her value is seen as dropping sharply. The ex-husband may not lose much*,* but for the woman*,* the cost is much greater.” (P15)*.

These accounts show that participants evaluated marriage not only through the possibility of entering a good relationship, but also through the difficulty of leaving a bad one. Legal procedures, divorce stigma, and the gendered consequences of motherhood made marriage appear riskier for women than for men. For these participants, the problem was not marriage itself, but the possibility that marriage could become difficult to exit while leaving women with heavier social, emotional, and financial costs.

#### Reproductive expectations exposed unequal bodily and future costs

Another major reason participants hesitated to enter marriage was that they associated it with fertility expectations and reproductive burdens that fell disproportionately on women. Here, marriage was not understood merely as couplehood, but as a package tied to childbirth, bodily sacrifice, and responsibility for children’s future well-being. Some participants linked this directly to broader social and economic conditions, questioning not only whether they wanted children, but whether the current environment justified having them at all.

*“Don’t blame me for not contributing to the increase of fertility rate. If they want me to have babies*,* they need to offer proof that my babies will have good lives. Based on the current economic conditions*,* I’m not seeing it happening.” (P5)*.

Others framed the issue through bodily knowledge and self-protection. Rather than accepting pronatalist persuasion as natural, they actively sought and circulated information about pregnancy and postpartum risks.

*“When I watch videos introducing potential harms during pregnancy and after giving birth on Xiaohongshu*,* I always share them with my family in our WeChat group*,* so that they can stop sharing those dumb videos persuading women to get married.” (P21)*.

These responses suggest that participants were not merely postponing marriage for career reasons or abstract self-development. They were also evaluating the reproductive dimension of marriage as one of its most unequal and least protected components. For many, the expectation that women should bear bodily risk, reproductive sacrifice, and long-term responsibility under uncertain economic conditions made marriage substantially less attractive.

#### Online misogyny weakened trust in men and marriage

At the broader public level, some participants connected their caution toward marriage to the wider online climate surrounding gender relations. What concerned them was not only the presence of misogynistic discourse, but also the fact that such views were sometimes endorsed or circulated by educated men, including male returnees.

*“I was shocked by how many people in my WeChat Moments were reposting Lao A’s views and comments*,* including male returnees. I did not expect such low-level incel rhetoric to receive likes and shares from people with higher education. As a result*,* I deleted everyone in my Moments who reposted or endorsed Lao A’s views.” (P20)*.

This concern was echoed by several participants, and this shows that online misogyny affected participants’ trust not only because of the original comments, but because of their circulation among men whom participants expected to be more reflective or respectful. For her, this made heterosexual partnership appear less trustworthy.

Another participant described how online discussion of women’s relationships with foreign men generated a particularly hostile form of resentment.

*“I saw social-media posts where women shared their experiences with foreign boyfriends and compared them with their former Chinese boyfriends. The comments were full of remarks like*,* ‘No wonder they come back and refuse to marry—Western men have spoiled their tastes.’ I found this deeply disgusting. These men seem to think that if we do not marry foreign men*,* then we somehow ought to belong to them.” (P9)*.

This account suggests that digital hostility toward female returnees was not limited to moral judgment. It also reflected an entitled logic in which women’s transnational intimacy was interpreted as a symbolic loss to domestic men, while women themselves were treated as objects of claim rather than autonomous subjects. Even so, participants did not present themselves as permanently opposed to marriage. Rather, many adopted a conditional stance: they remained open to marriage in principle, but only under circumstances that would not compromise their autonomy, dignity, bodily safety, or future quality of life. Together, these accounts suggest that online hostility shaped how some participants evaluated future relationships. Public comments about female returnees, foreign partners, and women’s sexual choices made marriage appear less like a secure intimate relationship and more like a setting in which women might face judgment, entitlement, or disrespect.

## Discussion

This study examined the experiences of single Chinese female returnees in post-COVID-19 China. The findings suggest that their reintegration difficulties are neither reducible to generic returnee adjustment nor fully captured by the domestic literature on unmarried professional women in China. Instead, participants’ experiences were shaped by the intersection of return migration, gendered social expectations, and marriage-related norms in a context marked by post-COVID uncertainty and intensified public scrutiny. Across the data, reacculturation-related distress emerged as a multi-layered process spanning family pressure, workplace inequality, and broader societal discourse, while participants’ orientations toward marriage reflected not a simple rejection of intimacy, but a conditional refusal to enter marriage unless it offered sufficient autonomy, dignity, safety, and reciprocity.

### Gendered reacculturation across family, workplace, and public life

The findings for RQ1 show that participants’ distress was distributed across multiple ecological layers rather than concentrated in any single domain. This supports prior returnee research suggesting that post-return adaptation is structured by interactions between interpersonal, institutional, and sociocultural environments rather than by intrapersonal adjustment alone [3, 4, 50]. In this respect, the present study reinforces the usefulness of an ecological perspective for understanding reacculturation among Chinese returnees, while extending it by showing how these pressures are specifically gendered for unmarried women.

At the mesosystem level, participants described family as a major source of strain, particularly through pressure surrounding marriage timing, gendered expectations, and socially approved life choices. This finding is broadly consistent with the domestic literature on *sheng nü*, which has shown that unmarried professional women in China often encounter intense parental pressure and are positioned within competing demands of modern achievement and traditional gender conformity [24–26]. However, the present study extends this literature by showing that, for female returnees, family conflict was not only about delayed marriage. It was also about the difficulty of reconciling overseas-shaped individual aspirations with kin expectations for stability, obedience, and a family-oriented life path. In this sense, family became not merely a site of ordinary marital pressure, but an arena in which returnees’ transnational identities were re-evaluated and, at times, disciplined based on family expectations.

At the exosystem level, participants’ accounts of workplace reintegration reveal a combination of sexual harassment, gender stereotyping, and restricted professional mobility. The finding that returnee women encountered glass ceilings and faced limited upward mobility in the absence of parental support aligns with earlier studies documenting the importance of parental social networks and social capital in career development [4, 51]. More importantly, while workplace sexism and harassment are well documented across diverse settings [52–56], the present findings suggest a more specific mechanism: participants described being treated as though overseas experience implied loosened sexual boundaries or greater tolerance of pornographic humor, unwanted physical intimacy, and other inappropriate conduct. In other words, they encountered both explicit and implicit forms of sexual harassment that were often disguised by aggressors as “cultural misunderstanding”. This is a novel finding and an important extension of existing returnee and feminist research, as it highlights women’s gendered reacculturation experiences when moving across societies with different gender norms, workplace cultures, and expectations surrounding women’s conduct.

At the macrosystem level, participants’ distress was amplified by what they perceived as a wider culture of gendered public hostility, moral judgment, and public devaluation of women. This resonates with existing work on gendered public discourse in China, including studies showing that media and online culture often stigmatize unmarried women and reproduce narrow ideals of femininity [25, 27]. However, the present study again suggests an added layer for returnee women. Participants did not describe only generalized sexism. They also described a public environment in which women associated with transnational mobility or intimacy with foreign men could become targets of sexualized and nationalist resentment. This is significant because it shows that public discourses linking women’s intimate choices to national or cultural loyalty intensified participants’ post-return pressures [37–39]. Whereas domestic single women are often stigmatized through delayed marriage and femininity norms, female returnees may also be judged through suspicion of Westernization, foreign ties, and symbolic distance from national-cultural loyalty. In this respect, the study contributes a more context-sensitive account of reacculturation.

### Marriage negotiation and the refusal of unequal terms

The findings for RQ2 suggest that participants did not approach marriage as a neutral or purely private life choice. Rather, they evaluated it as a gendered institution whose terms were progressively devalued by the layered pressures identified in RQ1. This is where the framework of PB is especially useful. Earlier studies on Chinese unmarried professional women have shown that delayed marriage is often not a simple rejection of convention, but a negotiated response to competing pressures and structurally unequal expectations [24, 26]. The present study builds on that insight by showing that, for female returnees, this negotiation is further shaped by reacculturative strain and transnational comparison.

First, participants’ narratives indicate that marriage still retained symbolic and practical value, but no longer appeared compelling on unequal terms. Some acknowledged that marriage could reduce parental pressure, offer access to additional resources, or provide companionship. This is in alignment with earlier studies on female Chinese returnees’ reacculturation experience [4, 57]. Yet these potential advantages were repeatedly weighed against the perceived costs of entering a low-quality or inequitable relationship. This is important because it distinguishes the present findings from a simplistic narrative of anti-marriage individualism [58, 59]. Participants were not uniformly rejecting marriage in principle; rather, they were refusing to enter marriage under conditions they considered degrading, unsafe, or emotionally costly. This is precisely the kind of conditional and strategic positioning that PB helps illuminate [49].

Second, the findings suggest that marriage was interpreted in relation to broader legal, sociocultural, and political conditions, rather than just interpersonal preference. Participants’ concern with the divorce “cooling-off period,” for example, indicates that women evaluated marriage through the lens of exit constraints as well as entry benefits. This extends existing discussions of Chinese women’s marriage negotiation by highlighting how local-provincial level legal interpretations and practices may alter the perceived terms of the marital bargain. In other words, in the current study, it is suggested that if leaving an unsatisfactory or harmful marriage appears procedurally and socioculturally difficult, then marriage itself becomes a riskier institutional commitment. In this sense, participants’ hesitancy was not merely emotional caution; it was a structurally informed reading of vulnerability.

Third, participants’ accounts linked marriage to reproduction in ways that exposed the asymmetry of women’s expected sacrifice. The fertility quote from P5 and the pregnancy-risk account from P21 are especially revealing, as both indicate resistance to the assumption that women should absorb reproductive, bodily, and future-oriented risks simply because women’s reproductive choices are sometimes framed as part of broader family, social, or demographic responsibility. This moves the analysis beyond the domestic *sheng nü* literature in an important way. The issue was not only delayed marriage, but also a refusal to accept pronatalist expectations under conditions of economic insecurity, inadequate support, and unequal bodily burden. This aspect of the findings is especially significant because it suggests that some participants were not merely resisting patriarchal marriage norms, but also pushing back against broader public discourses that link women’s reproductive choices to collective or demographic responsibility [60–63].

Finally, the study shows that digital misogyny and public hostility further eroded trust in men and reduced marriage’s perceived desirability. Participants’ responses to *Lao A*’s stigmatization of Chinese female international students are revealing not only because they show offense at misogynistic discourse, but also because they demonstrate the collapse of interpersonal trust that followed from it. For some participants, what became intolerable was not only *Lao A’*s rhetoric itself, but the extent to which other men, including returnee men, appeared willing to endorse it. Such misogynistic attitudes among Chinese male international students and returnees have already been observed in online communities [64], and *Lao A*’s inflammatory discourse appeared to reactivate and legitimize this hostility by casting Chinese female international students as morally suspect and sexually degraded. These gender confrontations observed in this study have been well-documented in recent studies [65, 66]. Furthermore, participants’ reactions to social-media comments about women who had dated foreign men are especially important here. These responses show that online hostility did not merely moralize women’s sexuality; it also reproduced a gendered-nationalist logic of possession and objectification, in which female returnees were treated as though they should remain available to domestic men rather than act as autonomous subjects. As this hostility circulated through social media, it further weakened female returnees’ trust in domestic men, thereby making heterosexual partnership appear less respectful, less trustworthy, and less emotionally safe.

### Implications

One of the major contributions of the study lies in its ability to bring together literature that are too often treated separately. Existing domestic feminist work on Chinese unmarried women has richly documented parental pressure, stigma, and marriage-market constraints [24–26], while returnee research has examined identity conflict, discrimination, and post-return strain [4, 5, 10]. The present study contributes by showing that these are not parallel processes for single female returnees. Rather, they intersect and mutually intensify one another. This intersectional positioning helps explain why female returnees may experience reacculturation not only as a process of coming home, but also as a process of re-entering a gendered and, at times, gender-nationalized social order.

Furthermore, the study has broader social significance. In a period marked by demographic anxiety, pronatalist pressure, and intensified digital hostility toward women, the experiences of single female returnees reveal how marriage, reproduction, and national belonging can become intertwined in ways that heighten women’s reacculturative burden. Their narratives suggest that improving women’s reintegration requires more than economic opportunity alone. It also requires confronting the gendered family expectations, workplace inequalities, and public discourses that continue to make women’s value conditional on conformity to unequal norms.

Finally, the findings also have implications for women’s participation in public life. Participants’ accounts show that the pressures surrounding single female returnees are not reducible to private family matters; rather, they are reproduced through workplaces, institutional arrangements, and public discourse. These gendered conditions may discourage women’s equal participation in professional and civic life by making visibility, advancement, and autonomy more psychologically costly. From a public health standpoint, this suggests that policies aimed at women’s well-being should move beyond individual coping and address the broader institutional and discursive environments that shape women’s public legitimacy, occupational mobility, and social participation.

### Limitations and future research

Several limitations should be acknowledged. First, this study is based on a relatively small qualitative sample of 22 single Chinese female returnees, recruited through purposive sampling via personal networks and WeChat returnee groups. Although this strategy was appropriate for accessing a population whose experiences are socially sensitive and not easily observable, it may also have introduced selection bias. Participants who were more reflective, more dissatisfied with their reacculturation, or more willing to speak about gendered pressures may have been more likely to participate. Accordingly, the findings should not be interpreted as statistically representative of all single Chinese female returnees. Future research would also benefit from large-scale, targeted survey studies of single female returnees that test the generalizability of the themes identified here (multi-layered acculturative stress, patriarchal bargaining around marriage, and the role of gendered nationalism) and examine whether similar patterns hold across broader occupational, regional, and socioeconomic strata.

Second, the sample was intentionally limited to never-married women who had studied in Western countries and returned to China. This focus strengthened the analytical coherence of the study, but it also narrowed its scope. The findings therefore cannot be readily generalized to male returnees, married female returnees, divorced returnees, or women returning from non-Western destinations. Nor can they fully capture regional, class-based, or sectoral variation within the broader returnee population. Although participants came from a range of industries and educational backgrounds, future research should examine whether similar patterns emerge among returnees situated in different occupational fields, family backgrounds, and localities.

Third, the study relies primarily on self-reported interview data. This allows access to participants’ lived meanings, but it also means that the analysis is based on narrated experiences rather than direct observation of family interactions, workplace practices, or online behaviors. Some accounts may be shaped by retrospective interpretation, emotional salience, or selective recall. At the same time, because the topic concerns stigma, harassment, and intimate decision-making, participants may also have withheld certain experiences or framed them cautiously. Future studies could strengthen the evidence base by triangulating interviews with additional materials, such as digital ethnography, diary-based methods, policy analysis, or comparative media/discourse analysis.

Fourth, although the present study captures participants’ interpretations at an important post-return moment, reacculturation and marriage negotiation are not static processes. Women’s views on intimacy, fertility, family obligation, and future planning may shift over time in response to changing employment conditions, family pressures, public controversies, or new relationships. For this reason, future research would benefit from longitudinal designs that follow returnees across different stages of reintegration. Repeated interviews, panel-based qualitative studies, or mixed-method designs could better capture how women’s positions toward marriage and singlehood evolve rather than treating them as fixed attitudes.

## Supplementary Information

Supplementary Material 1.

Supplementary Material 2.

## Data Availability

Given the sensitive nature of the study, the data that support the findings of this study will not be shared or disclosed.
