# Supplementary material for: Gendered reacculturation and marriage negotiation among single Chinese female returnees
Source: BMC Public Health. 2026 Jun 23;26:2530. doi: 10.1186/s12889-026-28227-x (PMC13543345; doi:10.1186/s12889-026-28227-x)
Supplement: Supplementary file 2 [file 12889_2026_28227_MOESM2_ESM.docx]

**Interview Guide**

1. Could you briefly tell me about your educational and overseas experience, and what your life looked like before you returned to China?
2. What initially influenced your decision to return to China, and how did you imagine life would be after returning?
3. Looking back on your return, how would you describe your adjustment during the first period after coming back?
4. In your everyday life since returning, what kinds of situations have felt easy, difficult, or unexpected for you?
5. How would you describe your current relationship with your family since returning to China?
6. Have there been any conversations or situations with family members that made you think differently about your life priorities, future plans, or personal choices?
7. How would you describe your experience in the workplace since returning?
8. Have there been any moments at work when you felt especially comfortable, uncomfortable, respected, misunderstood, or constrained?
9. In what ways, if any, do you think your overseas background has shaped how other people see or treat you in professional settings?
10. In your view, what kinds of messages or expectations do women like you encounter in society more broadly after returning to China?
11. Are there any media discussions, online conversations, or public events in recent years that have particularly stayed with you? What stood out to you about them?
12. At this stage in your life, how do you think about marriage?
13. When you think about the possibility of marriage, what factors make it seem more appealing, less appealing, or more complicated for you?
14. How do you think about issues such as childbirth, family formation, or future planning, and how do these considerations shape your views about intimate relationships or marriage?

*Follow-up questions diverged based on participants’ responses
